# Responses of retinal and brain microvasculature to streptozotocin induced diabetes revealed by global expression profiling

**DOI:** 10.1177/14791641221147533

**Published:** 2023-01-06

**Authors:** Youhai Li, Alen Faiz, Han Moshage, Lothar Schilling, Jan AAM Kamps

**Affiliations:** 1 Division of Neurosurgical Research, 9144 Heidelberg University, Mannheim, Germany; European Center of Angioscience, Medical Faculty Mannheim, 9144 Heidelberg University, Mannheim, Germany; 2 Department of Pathology and Medical Biology, 10173 University Medical Center Groningen, Groningen, The Netherlands; 3 Department of Gastroenterology and Hepatology, 10173 University Medical Center Groningen, Groningen, The Netherlands

**Keywords:** Retina, brain, diabetes, microvasculature, gene expression profiling

## Abstract

This study aims to determine the effects of diabetes in the retinal and brain microvasculature through gene expression profiling. Twelve male Wistar rats were randomly divided into two groups: streptozotocin-induced diabetic rats and time-matched nondiabetic rats. The retinal microvessels (RMVs) and brain microvessels (BMVs) were mechanically isolated from individual rats. Differentially expressed genes (DEGs) in diabetic and nondiabetic microvessels were identified by cDNA microarrays analysis. In RMVs, we identified 43 DEGs, of which 20 were upregulated while 23 were downregulated by diabetes. In BMVs, 35 genes DEGs were identified, of which 22 were upregulated and 13 were downregulated by diabetes. Altered expression of the Nars, Gars, Mars, Iars, Yars, Bcl2, Nqo1, NR4A3, Gpd1, Stc1, Tsc22d3, Tnfrsf21 mRNA as observed in the microarray analyses, was confirmed by quantitative RT-PCR. The aminoacyl-tRNA synthetases (aaRSs) pathway in RMVs was significantly overrepresented as compared to BMVs. Our study demonstrates for the first time that in the brain microvasculature multiple compensatory mechanisms exists, serving to protect brain tissue from diabetic insults, whereas these mechanisms are not activated in the retinal microvasculature. This provides new insights as to why brain microvasculature is less susceptible to diabetes.

## Introduction

Diabetes is a progressive metabolic disease characterized by hyperglycemia due to absolute or relative (resistance) insulin deficiency, and the development of chronic vascular damage in retina, kidney and peripheral nerves.^1^ Over the past decades, multiple molecular mechanisms have been proposed to explain the pathogenesis of diabetic vascular injury, e.g., overproduction of reactive oxygen species (ROS) in mitochondria and NF-κB pathway activation in endothelial cells.^2,3^ Despite extensive research, until now there are no effective therapies for preventing diabetic vascular complications.^1^

Heretofore, most studies have focused on the highly susceptible organs in diabetes, such as retinae, kidney and peripheral nerves, to identify key molecular mechanisms of diabetic microvascular complications. Researchers claimed that the molecular imbalance between toxic and endogenous protective factors may be responsible for the pathogenesis of diabetic microvascular complications.^4^ A wealth of studies reported that brain microvasculature was less susceptible to diabetes compared to retinal microvasculature.^5–7^ However, the underlying molecular mechanisms involved in this difference is poorly understood.

Many research efforts to elucidate the molecular mechanisms of diabetic retinopathy (DR) by assessing the genomic and/or proteomic profiles of the entire retina tissue.^8–10^ However, these findings might be inaccurate for explaining the molecular mechanisms of diabetic vascular impairments because of the presence of an excess nonvascular tissue in whole organ analysis. Recently, we have established a new mechanical isolation method for both retinal and brain microvessels (RMVs and BMVs, respectively) from normal and diabetic rats, allowing us to extract highly purified microvessels.^11^ In this study, we characterized the transcriptional changes of RMVs and BMVs by direct comparison of isolated microvessels from diabetic and time-matched control rats. We hypothesized that diabetes induces substantially different gene expression patterns in these two types of microvessels even though they are of the same embryological origin. We further investigated the balance of possible adverse and beneficial factors and pathways in RMVs and BMVs. Thereafter, these expression profiles identified by microarray analysis were validated by quantitative RT-PCR while NR4A3 mRNA expression alteration in response to hyperglycemia was tested in *vitro*.

## Material and methods

### Animals

The animal study was approved by the Federal Animal Ethics Committee (Karlsruhe, Germany). All experimental procedures complied with the ethical regulations of the Directive 2010/63/EU. Type 1 diabetes mellitus was induced in male Wistar rats (9 weeks old; obtained from Janvier, Isle St- Genest, France) by a tail vein injection of Streptozotocin (35 mg/kg body weight, Sigma-Aldrich, Germany; prepared in 10 mM citrate buffer, pH 4.5) and confirmed by casual blood glucose ≥300 mg/dl. Age-matched control animals received vehicle injection. All animals were housed in cages with a 12-h light/dark cycle and given tap water and chow ad libitum. Three months after diabetes induction, the rats were deeply anesthetized with CO2 inhalation and then sacrificed. Eyes and brain hemispheres that removed meninges and associated vessels, were snapped frozen in liquid nitrogen and stored at −80°C until use.

### Isolation of brain and retinal microvessels

The rat RMVs and BMVs were isolated as described previously.^11^ Briefly, brain and retinal crysections were individually homogenized using a motor-driven homogenizer (Homgen plus, Schuett Biotec, Goettingen, Germany). The brain homogenate was centrifuged at 438g for 10min, followed by centrifugation at 4400g for 15min, after which the pellet was resuspended into 7 mL PBS/1% dextran (Dextran 70,000, Roth). Thereafter, the brain and retinal suspension were individually transferred onto a density gradient column and centrifuged for 15 min (1300 g). Finally, the microvessels were captured after filtration over a 60 µm nylon mesh. All the procedures were performed at 0°C.

### Human umbilical vein endothelial cells

Human umbilical vein endothelial cells (HUVECs) were purchased from Lonza (Lonza, Breda, The Netherlands) and maintained in EGM-2 media consisting of endothelial basal medium-2 supplemented with growth factors and antibiotics (EGM-2 SingleQuots kit, Lonza, The Netherlands). The effect of hyperglycemia on HUVECs was investigated by incubating cells in EGM-2 media with 30 mM D-glucose. The normal EGM-2 media used as a control contained 5.5 mM D-glucose. In all experiments, cells from passage 4 or 5 were used and cultured at 37°C in a 5% CO2/95% humidified atmosphere.

### RNA isolation and assessment

Total RNA was isolated from individual RMVs or BMVs using a RNeasy® Plus Micro kit (Qiagen, Hilden, Germany) according to the manufacturer’s instructions. The RNA concentration and integrity were assessed by Agilent 6000 Pico kit (RMVs and BMVs) on an Agilent 2100 bioanalyzer (Agilent Technologies, USA). Only samples with an integrity number (RIN) > 7.0 were used for microarray processing. For HUVECs, total RNA was isolated using a RNeasy® Plus Mini kit (Qiagen, Venlo, The Netherlands) according to the manufacturer’s instructions. The concentration and integrity of the RNA was determined using an ND-100 UV-Vis Spectrophotometer (Nanodrop Technologies, Rockland, DE, USA) and by agarose (1%) gel electrophoresis.

### Microarray processing and data analysis

Transcriptome profiles of the BMVs and RMVs were determined using the GeneChip® WT Pico Reagent Kit and the whole-transcriptome Rat Gene 2.0 ST array (Affymetrix, Inc., Santa Clara, CA, USA) as described previously.^12^ The raw CEL files were normalized using the Affymetrix® Expression Console Software (version 4.0, Affymetrix). The pairwise comparisons of nondiabetic and diabetic microvessels were performed with R software using LIMMA package (version 3.02; R Development Core Team, 2013). The obtained false discovery rate (FDR) adjusted *p* value and fold change (FC) were used as a cut-off to identify the differentially expressed genes (DEGs). Genes with a FDR adjusted *p* < 0.05 and FC >1.2 were considered statistically significant. The Kyoto Encyclopedia of Genes and Genomes (KEGG) pathways of DEGs were assessed using DAVID (https://david.ncifcrf.gov/summary.jsp). The complete microarray dataset is available at Gene Expression Omnibus (GEO) database (http://www.ncbi.nlm.nih.gov/geo/) under the accession number GSE113686

### Quantitative real-time PCR

Quantitative real-time PCR (qRT-PCR) was used for the confirmation of microarray data and for quantifying the expression level of Nr4a3 in HUVECs. Briefly, first-strand cDNA was generated from RNA samples by a 20 μL mixture containing SuperScript™ III RT (Invitrogen, Bleiswijk, the Netherlands), RNase Out inhibitor (40 units; Invitrogen) and 250 ng random hexamers (Promega, Leiden, the Netherlands). 10 ng of cDNA was used for each PCR reaction. Assays were performed on a ViiA 7 real-time PCR System (Applied Biosystems, Nieuwerkerk aan den IJssel, The Netherlands) using the absolute QPCR Rox Mix (Thermo Fischer Scientific). Samples were normalized with ΔCt method using GAPDH as a reference. Fold change in gene expression versus control was analyzed by the 2^−ΔΔCt^ method.^13^ Rat GAPDH (assay ID Rn01775763_g1), Nars (assay ID Rn01491242_m1), Mars (assay ID Rn01504657_m1), Yars (assay ID Rn01749701_m1), Gars (assay ID Rn01410234_m1), Iars (assay ID Rn01450644_m1), Bcl2 (assay ID Rn99999125_m1), Nqo1 (assay ID Rn00566528_m1), Nqo1 (assay ID Rn00566528_m1), NR4A3 (assay ID Rn01534012_m1), Gpd1 (assay ID Rn00573596_m1), Stc1 (assay ID Rn00579636_m1) and Tsc22d3 (assay ID Rn00580222_m1) were purchased as Assay-on-Demand from Applied Biosystems (Nieuwekerk a/d IJssel, the Netherlands).

### Statistics

Statistical analyses and graph plotting were carried out using GraphPad Prism 6.0 (GraphPad Prism Software Inc., CA, USA). Statistical differences were evaluated by Student’s *t*-test or ANOVA with post hoc comparison using Bonferroni correction. Data are given as mean ± SEM, unless stated otherwise. Differences were considered significant at *p* < 0.05.

## Results

**Figure 1.**
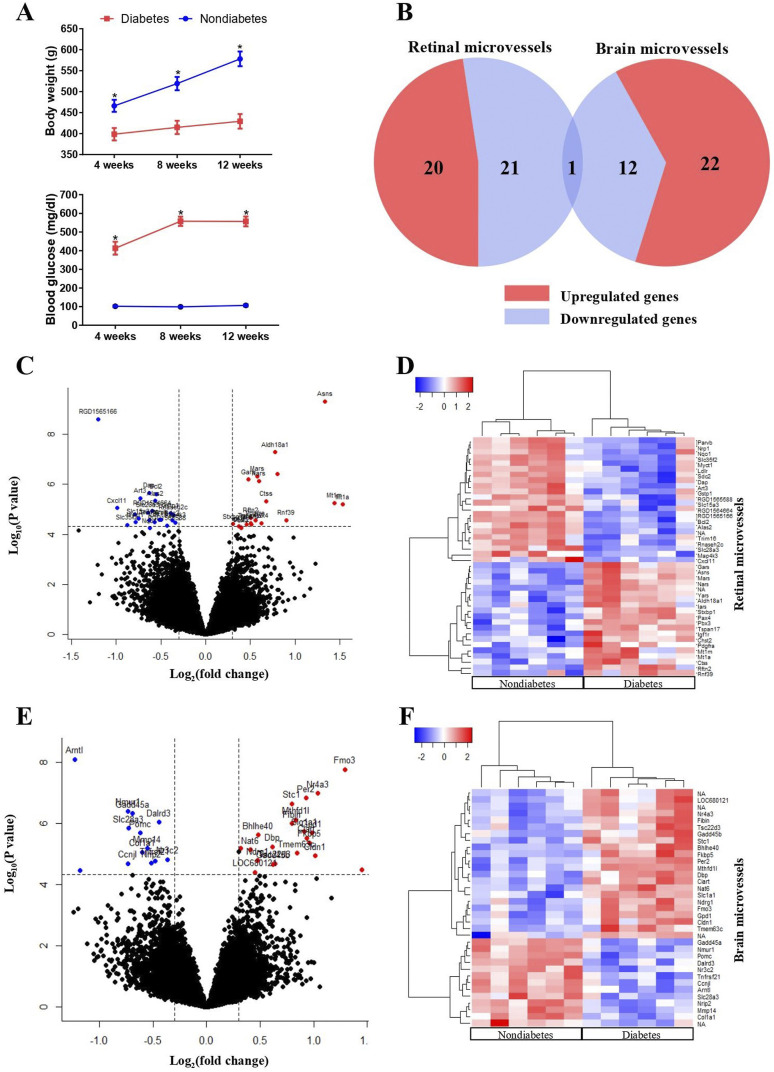
Responses of retinal and brain microvasculature to diabetes. (a) Average body weights and plasma glucose concentrations for diabetic (red line) and nondiabetic rats (blue line). Results are means ± SEM, *n* = 6. *Significantly different from non-diabetic animals. (b) Venn diagram for the significantly changed genes by diabetes in the retinal microvessels (RMV) and brain microvessels (BMVs). Volcano plots of all genes identified from RMVs (c) and BMVs (e). Significant differentially expressed genes (DEGs) are located between the vertical and horizontal dotted lines and are highlighted in red or blue. Heatmaps of the DEGs either from the RMVs (d) or the BMVs (f) are displayed.

The body weights and plasma glucose concentrations of the rats are displayed in Figure 1(a). The streptozotocin-induced diabetic rats show a significantly higher blood glucose level and significantly lower body weight compared to age-matched nondiabetic rats.

To investigate the diabetic effects on the gene expression profiles of retinal microvasculature and brain microvasculature, pairwise comparisons were performed: 1) Diabetic RMVs versus nondiabetic RMVs; and 2) Diabetic BMVs versus nondiabetic BMVs. Each group contained 6 non-pooled samples isolated from 6 individual rats. Microarray analysis was used as a discovery step, and the significantly differentially expressed genes (DEGs) were identified by a FDR adjusted *p* < 0.05 and FC >1.2 compared to nondiabetic samples.

### Differentially expressed genes in retinal and brain microvasculature

**Figure 2.**
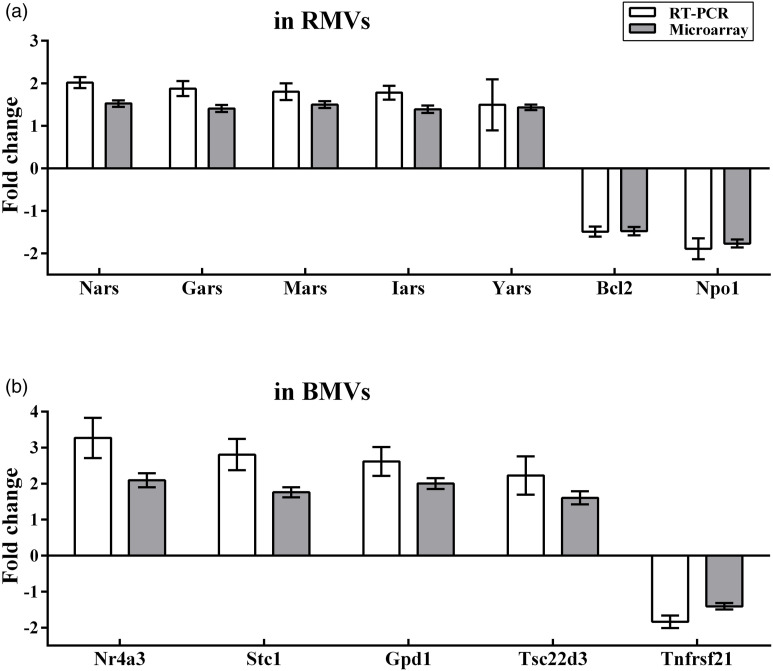
Validation of the microarray data by quantitative RT-PCR. Results for 12 genes are shown, of which seven genes are significantly changed by diabetes in RMVs (a), and five genes are significantly changed in BMVs (b). Microarray and qRT-PCR fold change values (2^−ΔΔCt^) were calculated for each gene for comparison between diabetic and nondiabetic samples. Results were shown as mean ± SD (n = 6).

In RMVs, 43 genes were significantly changed by the streptozotocin-induced diabetes (Figure 1(b)), of which 20 were upregulated and 23 were downregulated (Figure 1(c)). For the BMVs, 35 genes were significantly changed by the streptozotocin-induced diabetes (Figure 1(b)). Among these DEGs, 22 genes were upregulated and 13 were downregulated (Figure 1(e)). Hierarchical cluster analysis was performed for the DEGs from RMVs and BMVs as depicted in Figure 2(d) and (f), respectively. When we compared the DEGs of the RMVs and BMVs, only one gene (Slc28a3) overlapped (downregulated) between the RMVs and the BMVs (Figure 1(b)). In Table 1 and 2, the detailed information describing the upregulated and downregulated genes in RMVs and BMVs is displayed.

**Table 1.** Genes significantly changed by diabetes in the retinal microvessels.

Transcript ID	Gene symbol	Description	Diabetes versus control	
			Adj. P	FC
17,717,103	Mt1a	Metallothionein 1a	0.0168	2.91
17,752,700	Mt1m	Metallothionein 1M	0.0156	2.71
17,789,627	Asns	Asparagine synthetase	0.0000	2.51
17,756,041	Rnf39	Ring finger protein 39	0.0368	1.87
17,642,026	Aldh18a1	Aldehyde dehydrogenase 18 family, member A1	0.0006	1.72
17,740,865	Ctss	Cathepsin S	0.0145	1.60
17,790,133	Pax4	Paired box 4	0.0400	1.55
17,726,760	Nars	Asparaginyl-tR synthetase	0.0038	1.52
17,836,518	Mars	Methionyl-tR synthetase	0.0034	1.48
17,694,084	Pdgfra	PDGF receptor, alpha polypeptide	0.0368	1.47
17,802,403	Yars	Tyrosyl-tR synthetase	0.0408	1.42
17,718,250	Tspan17	Tetraspanin 17	0.0400	1.42
17,864,675	Rftn2	Raftlin family member 2	0.0368	1.42
17,791,502	Gars	Glycyl-tR synthetase	0.0038	1.39
17,718,501	Iars	Isoleucyl-tR synthetase	0.0408	1.38
17,854,693	Chst2	Carbohydrate sulfotransferase 2	0.0485	1.32
17,771,507	Pbx3	Pre-B-cell leukemia homeobox 3	0.0488	1.32
17,617,445	Igf1r	Insulin-like growth factor 1 receptor	0.0435	1.29
17,771,351	Stxbp1	Syntaxin-binding protein 1	0.0408	1.23
17,819,788	Map4k3	Mitogen-activated protein kinase kinase kinase kinase 3	0.0395	−1.27
17,623,163	Rnaseh2c	Ribonuclease H2, subunit C	0.0301	−1.28
17,828,801	Sdc2	Syndecan-2	0.0368	−1.29
17,639,089	Gstp1	Glutathione S-transferase pi 1	0.0285	−1.31
17,788,530	RGD1565588	Similar to calcium binding protein P22	0.0431	−1.35
17,731,247	Nrp1	Neuropilin-1	0.0368	−1.41
17,831,256	Parvb	Parvin, beta	0.0368	−1.43
17,683,445	Bcl2	B-cell CLL	0.0100	−1.46
17,871,623	Alas2	5-Aminolevulite synthase 2	0.0145	−1.47
17,842,577	Ldlr	Low density lipoprotein receptor	0.0395	−1.48
17,774,787	RGD1564664	Similar to LOC387763 protein	0.0261	−1.52
17,733,363	Nqo1	D(P)H dehydrogenase, quinone 1	0.0490	−1.54
17,736,949	Dap	Death-associated protein	0.0100	−1.55
17,646,726	Trim16	Tripartite motif-containing 16	0.0368	−1.55
17,714,143	Slc28a3	Solute carrier family 28, member 3	0.0285	−1.57
17,693,364	Art3	ADP-ribosyltransferase 3	0.0130	−1.66
17,624,116	Slc15a3	Solute carrier family 15, member 3	0.0368	−1.68
17,611,535	Myct1	Myc target 1	0.0395	−1.72
17,844,192	Slc35f2	Solute carrier family 35, member F2	0.0409	−1.83
17,688,703	Cxcl11	Chemokine (C-X-C motif) ligand 11	0.0215	−1.99
17,643,730	RGD1565166	Similar to MGC45438 protein	0.0000	−2.30

Adj. P, a false discovery rate (FDR) adjusted *p* value; FC, fold change.

**Table 2.** Genes significantly changed by diabetes in the brain microvessels.

Transcript ID	Gene symbol	Description	Diabetes versus control	
			Adj. P	FC
17,686,071	Fmo3	Flavin containing monooxygenase	0.0003	2.45
17,798,725	Nr4a3	Nuclear receptor subfamily 4, group A, member 3	0.0013	2.06
17,666,537	Cldn1	Claudin 1	0.0169	2.03
17,832,266	Gpd1	Glycerol-3-phosphate dehydrogenase 1 (soluble)	0.0055	1.97
17,757,188	Fkbp5	FK506 binding protein 5	0.0092	1.95
17,749,555	Ciart	Circadian associated repressor of transcription	0.0070	1.91
17,866,453	Per2	Period circadian clock 2	0.0014	1.91
17,624,812	Slc1a1	Solute carrier family 1 member 1	0.0055	1.88
17,817,653	Tmem63c	Transmembrane protein 63c	0.0145	1.80
17,611,443	Mthfd1l	Methylenetetrahydrofolate dehydrogenase (NADP + dependent) 1-like	0.0036	1.79
17,699,814	Stc1	Stanniocalcin-1	0.0017	1.73
17,775,237	Fibin	Fin bud initiation factor homolog (zebrafish)	0.0038	1.73
17,879,047	Tsc22d3	TSC22 domain family, member 3	0.0247	1.56
17,833,546	Gadd45b	Growth arrest and D-damage-inducible, beta	0.0248	1.54
17,616,470	Dbp	D site of albumin promoter (albumin D-box) binding protein	0.0118	1.54
17,785,849	Bhlhe40	Basic helix-loop-helix family, member e40	0.0059	1.39
17,837,791	Ndrg1	N-myc downstream regulated 1	0.0222	1.39
17,664,242	LOC680121	Similar to heat shock protein 8	0.0421	1.37
17,847,497	Nat6	N-acetyltransferase 6 (GCN5-related)	0.0128	1.33
17,732,995	Nr3c2	Nuclear receptor subfamily 3, group C, member 2	0.0216	−1.27
17,847,732	Dalrd3	DALR anticodon binding domain containing 3	0.0038	−1.28
17,863,116	Tnfrsf21	Tumor necrosis factor receptor superfamily, member 21	0.0222	−1.29
17,787,500	Nrip2	Nuclear receptor interacting protein 2	0.0247	−1.31
17,698,634	Mmp14	Matrix metallopeptidase 14 (membrane-inserted)	0.0122	−1.35
17,650,326	Col1a1	Collagen, type I, alpha 1	0.0140	−1.41
17,814,505	Pomc	Proopiomelanocortin	0.0055	−1.43
17,791,733	Gadd45a	Growth arrest and D-damage-inducible, alpha	0.0025	−1.46
17,714,143	Slc28a3	Solute carrier family 28, member 3	0.0049	−1.47
17,645,118	Ccnjl	Cyclin J-like	0.0247	−1.48
17,866,181	Nmur1	Neuromedin-U receptor 1	0.0025	−1.52
17,619,710	Arntl	Aryl hydrocarbon receptor nuclear translocator-like	0.0003	−1.54

Adj. P, a false discovery rate (FDR) adjusted *p* value; FC, fold change.

### Biological pathways

Among the 43 genes of RMVs that were changed under diabetic conditions, the DAVID functional annotation analysis identifies three enriched KEGG pathways: (i) the aminoacyl-tRNA biosynthesis pathway, including Iars, Yars, Nars, Gars and Mars; (ii) the focal adhesion pathway, including Igf1r, Pdgfra, Bcl2 and Parvb; and (iii) the prostate cancer pathway including Igf1r, Pdgfra and Bcl2 (Table 3). In BMVs, the circadian rhythm pathway including Per2, Bhlhe40 and Arntl, was significantly enriched (Table 3).

**Table 3.** Pathway identification among the diabetes changed genes in RMVs and BMVs.

KEGG pathways	Gene symbols	*p* value	Fold change
In RMVs			
Aminoacyl-tRNA biosynthesis	Iars, yars, nars, gars, mars	5.E−05	23
Focal adhesion	Igf1r, pdgfra, Bcl2, parvb	3.E−02	6
Prostate cancer	Igf1r, pdgfra, Bcl2	3.E−02	11
In BMVs			
Circadian rhythm	Per2, Bhlhe40, arntl	2.E−03	43

The Kyoto Encyclopedia of Genes and Genomes (KEGG) pathways were obtained using DAVID Functional Annotation tool. RMVs, retinal microvessels; BMVs, brain microvessels.

### Validation of the microarray data with qRT-PCR

To confirm the outcome of the microarray analyses, quantitative real-time PCR (qRT-PCR) analyses were performed on the same RNA samples as used in the microarray studies. Seven DEGs (Yars, Mars, Iars, Nars, Gars, Bcl2 and Nqo1) from RMVs and five DEGs (NR4A3, Stc1, Gpd1, Tsc22d3 and Tnfrsf21) from BMVs were selected. For individual altered genes of RMVs or BMVs, results obtained by qRT-PCR are consistent with the microarray findings, in terms of direction and extent (Figure 2). Overall, there is a good and statistically significant correlation (r = 0.905, *n* = 24) between the magnitude of altered expression measured by microarray and expression measured by qRT-PCR.

### Hyperglycemia induced NR4A3 gene upregulation

**Figure 3.**
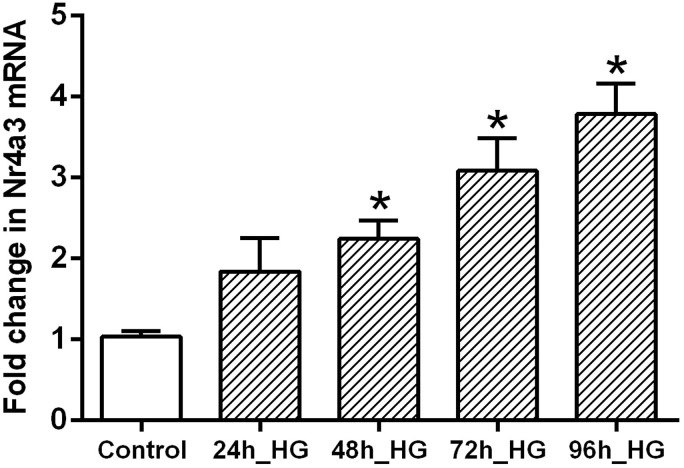
The effect of high glucose on NR4A3 gene expression in human umbilical vein endothelial cells (HUVECs). Cells were cultured with 5.5 mm (control) or 30 mm (HG) D-glucose for 96 h. The HG-treated cells were divided into four distinct groups based on the duration of the high glucose exposure. Experiments were repeated in triplicates with different cell preparations. mRNA fold change is relative to controls while using GAPDH expression as a reference. Results are given as mean ± SEM, *n* = 9. *Significantly different from the control.

It has been reported that NR4A3 is involved in glucose metabolism and NF-κB pathway activation.^14–16^ Under diabetic conditions, expression of NR4A3 mRNA was significantly increased in BMVs, as measured by microarray and qRT-PCR (Figure 2). NR4A3 mRNA was identified as the gene with the biggest difference between RMVs and BMVs (fold change of 2.4 and *p* < 0.0001). In HUVECs, NR4A3 gene expression was significantly increased by high glucose in a time-dependent manner (Figure 3).

## Discussion

This is the first report in which the effects of diabetes on the gene expression pattern of rat retinal microvasculature were analyzed by comparing the whole transcriptome between diabetic and nondiabetic RMVs. In parallel, the effects of diabetes on the gene expression pattern of rat brain microvasculature were also analyzed. This study shows that in diabetes, BMVs have different gene expression patterns compared to RMVs (e.g., the aminoacyl-tRNA synthetases), which allow for identification of novel targets for protective vascular intervention strategies.

High glucose-induced ROS overproduction has been considered as the principal cause of diabetic microvascular damage.^2,17,18^ Although cerebral endothelial cells are also exposed to abnormally high glucose concentrations, the brain microvasculature is not noticeably changed. The underlying mechanisms are far from clear. In the present study, we observe that in diabetes, expression of NAD(P)H dehydrogenase 1 (Nqo1) and glutathione S-transferase P (Gstp1) is significantly downregulated in RMVs (Table 1) while it is not changed in BMVs. It is well documented that that both Nqo1 and Gstp1, enzymes with antioxidant activity, are pivotal in the intracellular defense mechanisms to counteract ROS productions.^19–21^ In addition, Stanniocalcin-1 (Stc-1) and Bhlhe40, enzymes that suppress superoxide generation and hence protect cells from ROS-induced damage,^22–25^ are significantly overexpressed in BMVs (Table 2). These findings suggest that, in diabetes the compensatory/protective capacity in BMVs appear to be enhanced by expression of genes that code for antioxidants, whereas these are suppressed in RMVs.

Methylglyoxal (MG), a major precursor of advanced glycation end products (AGEs), is highly toxic to tissue and is considered as an important cause of diabetic complications.^26,27^ Its primary source is dihydroxyacetone phosphate (DHAP) that is an isomer of glyceraldehyde 3 - phosphate (GAP). Previous studies have shown that hyperglycemia-induced oxidative stress leads to DNA damage and activation of nuclear poly(ADP-ribose) polymerase (PARP, a nuclear DNA repair enzyme), which inhibits the catalytic activity of glyceraldehyde 3-phosphate dehydrogenase (GAPDH).^28,29^ It is suggested that as a result the levels of glycolytic metabolites (e.g., MG, GAP and DHAP) that are upstream of GAPDH increase, resulting in activation of multiple pathogenic pathways in diabetes, such as activation of protein kinase C (PKC) and an increase of AGEs. In the present study, we observe that in diabetes, expression of glycerol-3-phosphate dehydrogenase 1 (Gpd1) is significantly upregulated in BMVs (Table 2) while it is not changed in RMVs. Gpd1 is a key enzyme that converts DHAP into glycerol-3-phosphate (G3P) with a decrease in the NADH/NAD^+^ ratio.^30^ This process can reduce cellular concentration of DHAP and prevent the spontaneous conversion of DHAP into MG.^31^ The overexpression of Gpd1 in BMVs may protect the brain microvasculature against toxic glycolytic metabolites-induced injuries.

High glucose-induced activation of the nuclear factor (NF)-κB pathway in vascular cells is also a key contributor to the pathogenesis of diabetic complications.^4,32–34^ Here, we observe that in diabetes the expression of Tnfrsf21 (TNF receptor superfamily member 21) in BMVs is significantly decreased by diabetes (Table 2). Tnfrsf21 activates the NF-κB pathway and triggers cell apoptosis.^35^ In addition, we further observe that in diabetes, expression of neuron-derived orphan receptor 1 (Nr4a3) and TSC22 domain family protein 3 (Tsc22d3) in BMVs is significantly increased (Table 2) while it is not changed in RMVs. It has been demonstrated that both Nr4a3 and Tsc22d3 exhibit anti-apoptotic effects through prevention of NF-κB pathway activation.^14,36,37^ Expression of Nr4a3 plays also a critical role in neuronal protection.^38,39^ Badrichani AZ, et al. reported that expression of B-cell lymphoma 2 (Bcl-2, an important anti-apoptotic protein) protects endothelial cells from TNF-induced apoptosis through inhibition of the NF-κB pathway.^40^ In diabetes, expression of Bcl-2 in RMVs is significantly decreased (Table 1) while it is not changed in BMVs. The anti-inflammatory mechanism in BMVs seems to be enhanced by inhibition of the NF-κB pathway whereas it might be suppressed in RMVs.

Aminoacyl-tRNA synthetases (aaRSs) catalyze the ligation of amino acids to their cognate tRNAs, thereby playing a crucial role in protein synthesis.^41^ In addition, many studies have shown that aaRSs also have multiple noncanonical functions including regulation of glucose metabolism, angiogenesis, inflammation and cell stress responses;^42–45^ and aberrant expression or variants of aaRSs are involved in various diseases.^46–48^ In this study, we found that the expression of Nars, Gars, Mars, Iars and Yars (5 components of aaRSs) in RMVs were significantly upregulated in diabetes, whereas these genes were not changed in BMVs. Previous studies have shown that oxidative stress can cause damage to aaRSs functions, followed by amino acid mistranslation and protein misfolding.^49,50^ We speculate that the upregulation of aaRSs genes in RMVs is caused by the hyperglycemia-induced ROS overproduction, which in turn affects the reliability of protein translation in RMVs.

Clearly, functional and mechanistic studies are necessary to substantiate the complex processes and the precise effects of the discussed gene expression patterns in BMV and RMV in diabetes. Nevertheless, our study suggests that BMVs have defense mechanisms including reduction of ROS production, reduction of glycolytic intermediates and enlarged anti-inflammatory capacity, against the detrimental effects of diabetes. In contrast, in RMV these protective systems are not activated or even suppressed, resulting in a diminished ability to balance the potentially toxic factors that are induced by diabetes. These findings will increase our knowledge and understanding of the mechanisms playing a role in the different susceptibilities to diabetes of microvessels in retina and brain and may pave the way to the discovery of novel treatments to intervene in diabetic-induced microvascular complications.
